# Supplementary material for: The state of OA: a large-scale analysis of the prevalence and impact of Open Access articles
Source: PeerJ. 2018 Feb 13;6:e4375. doi: 10.7717/peerj.4375 (PMC5815332; doi:10.7717/peerj.4375)
Supplement: Supplemental Information 1 [file peerj-06-4375-s001.docx]

## Appendix: Additional analyses

## How does Open Access vary by repository?

Which repositories host the papers that are available only through repositories? We looked at all papers published between 1950 and 2015, and found that PMC is most common, hosting almost a third of all such content, as shown in Table A1.

| Repository | n | percent |
| --- | --- | --- |
| PMC | 1315 | 31.0 |
| ArXiv | 651 | 15.4 |
| domain ending in .edu | 1161 | 27.4 |
| other | 1110 | 26.2 |

**Table A1: Proportion of articles with OA copies found from a sample of articles published since 2009, by publisher.**

Furthermore, the role of PMC is even more dominant for more recently published papers, relative to other repositories, as seen in Figure A1.


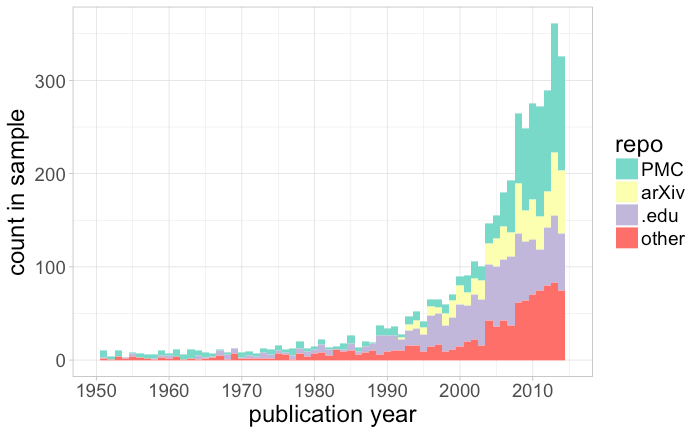


**Figure A1: Number of articles found only in repositories from a sample of 100,000 journal articles, by year of publication, as measured in 2017.**

Analyses performed in the main paper have considered papers in repositories only when they are not available for free on the publisher site. Figure A2 and A3 remove this restriction, counting all papers that have been deposited into repositories, regardless of Gold OA status. We can see that by this more permissive definition of Green, Green OA is more common for recently published papers in both absolute and proportional numbers.

###
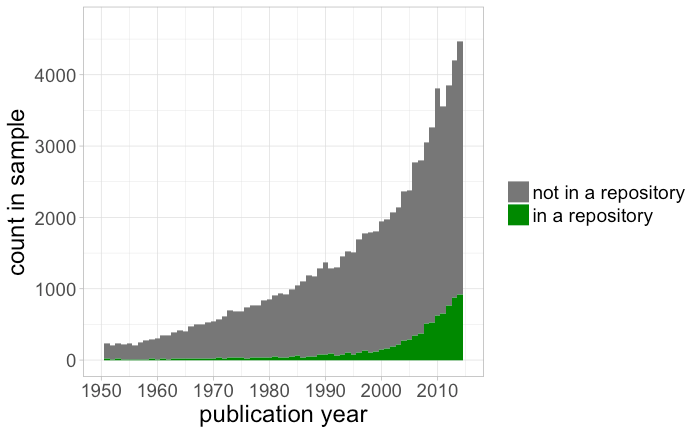


**Figure A2: Number of articles found in repositories from a sample of 100,000 journal articles, by year of publication, as measured in 2017.**

###
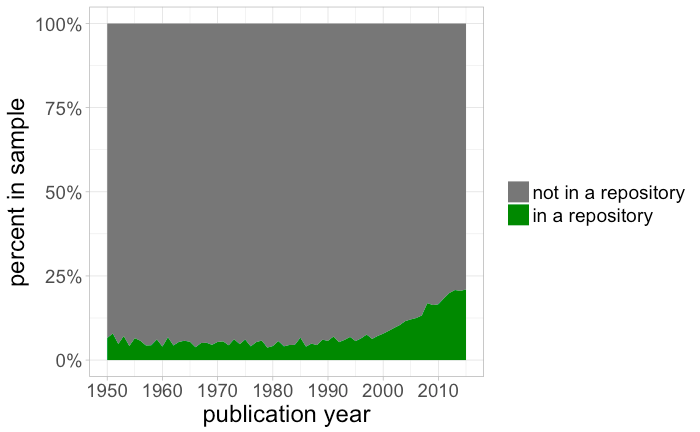


**Figure A3: Proportion of articles found in repositories from a sample of 100,000 journal articles, by year of publication, as measured in 2017.**

###

###

### What are the most common Open Access licenses?

We found a license for only 14.8% of open access articles. When a license was found, in more than half the cases it was CC-BY, a trend which increases slightly by year of publication, as shown in Figure A4.


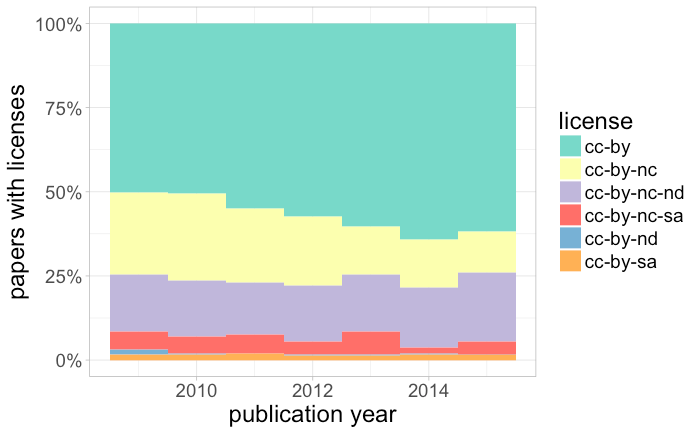


**Figure A4: Proportion of articles by license, for open access articles with a license found.**


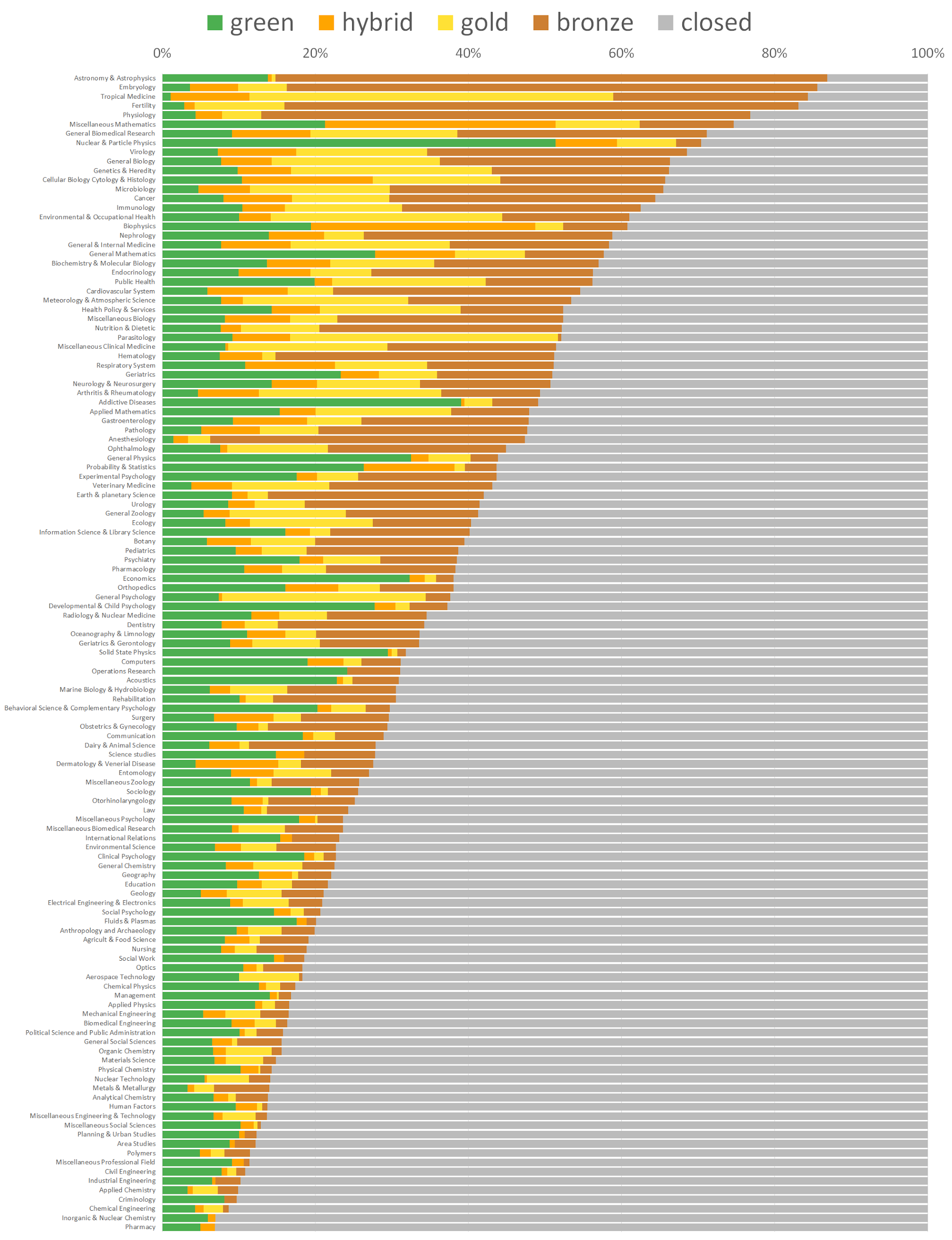


**Figure A5. Percentage of different access types of a random sample of WoS articles and reviews with a DOI published between 2009 and 2015 per NSF specialty (excluding Arts and Humanities and specialties with less than 100 papers).**
